# Methyl 4-acet­oxy-2-methyl-2*H*-1,2-benzothia­zine-3-carboxyl­ate 1,1-dioxide

**DOI:** 10.1107/S1600536808004029

**Published:** 2008-02-15

**Authors:** Matloob Ahmad, Hamid Latif Siddiqui, Saeed Ahmad, Muhammad Irfan Ashiq, Graham John Tizzard

**Affiliations:** a Institute of Chemistry, University of the Punjab, Lahore 54600, Pakistan; b Department of Chemistry, University of Science and Technology, Bannu, Pakistan; c School of Chemistry, University of Southampton, England

## Abstract

In the title compound, C_13_H_13_NO_6_S, the thia­zine ring adopts a distorted half-chair conformation. Each mol­ecule is linked to its neighbour through inter­molecular C—H⋯O hydrogen bonds.

## Related literature

For related literature, see: Fabiola *et al.* (1998 ▶); Golič & Leban (1987 ▶); Kojić-Prodić & Rużić-Toroš (1982 ▶); Rajagopal & Seshadri (1990 ▶); Reck *et al.* (1988 ▶); Rehman *et al.* (2005 ▶, 2006 ▶); Turck *et al.* (1996 ▶).
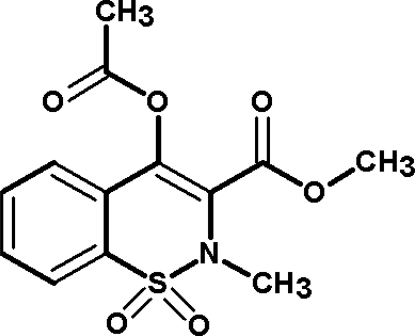


## Experimental

### 

#### Crystal data

C_13_H_13_NO_6_S
*M* *_r_* = 311.30
Monoclinic, 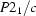

*a* = 6.8917 (5) Å
*b* = 24.1814 (17) Å
*c* = 8.2861 (5) Å
β = 97.876 (4)°
*V* = 1367.86 (16) Å^3^
*Z* = 4
Mo *K*α radiation
μ = 0.26 mm^−1^
*T* = 120 (2) K
0.40 × 0.20 × 0.20 mm

#### Data collection

Bruker Nonius KappaCCD diffractometer
Absorption correction: multi-scan (*SADABS*; Sheldrick, 2007 ▶) *T*_min_ = 0.902, *T*_max_ = 0.949
12265 measured reflections
3032 independent reflections
2183 reflections with *I* > 2σ(*I*)
*R*_int_ = 0.057

#### Refinement

*R*[*F* ^2^ > 2σ(*F* ^2^)] = 0.050
*wR*(*F* ^2^) = 0.145
*S* = 1.03
3032 reflections
193 parameters
H-atom parameters constrained
Δρ_max_ = 0.36 e Å^−3^
Δρ_min_ = −0.55 e Å^−3^

### 

Data collection: *COLLECT* (Nonius, 1998 ▶); cell refinement: *DENZO* (Otwinowski & Minor, 1997 ▶) and *COLLECT*; data reduction: *DENZO* and *COLLECT*; program(s) used to solve structure: *SHELXS97* (Sheldrick, 2008 ▶); program(s) used to refine structure: *SHELXL97* (Sheldrick, 2008 ▶); molecular graphics: *CAMERON* (Pearce & Watkin, 1993 ▶); software used to prepare material for publication: *WinGX* (Farrugia, 1999 ▶).

## Supplementary Material

Crystal structure: contains datablocks I, global. DOI: 10.1107/S1600536808004029/kp2156sup1.cif

Structure factors: contains datablocks I. DOI: 10.1107/S1600536808004029/kp2156Isup2.hkl

Additional supplementary materials:  crystallographic information; 3D view; checkCIF report

## Figures and Tables

**Table 1:** Hydrogen-bond geometry (Å, °)

*D*—H⋯*A*	*D*—H	H⋯*A*	*D*⋯*A*	*D*—H⋯*A*
C13—H13*A*⋯O4^i^	0.98	2.48	3.387 (3)	153
C5—H5⋯O1^i^	0.95	2.48	3.349 (3)	151

Symmetry code: (i) 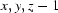.

**Fig. 1.**
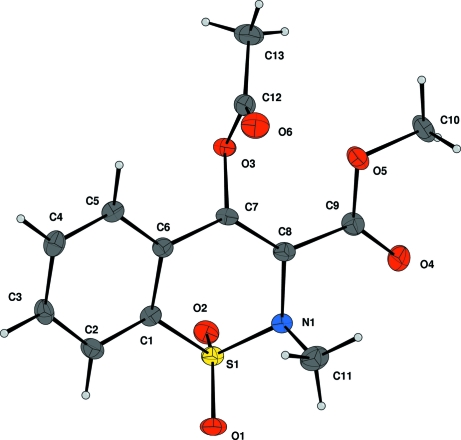
The molecular structure of (I), showing displacement ellipsoids at the 50% probability level for non-H atoms. Dashed lines denote hydrogen bonds.

## supplementary crystallographic information

### Comment

Among the vast class of benzothiazines, 1,2-benzothiazine1,1-dioxides are the most versatile compounds due to their applications in various fields such as pharmaceuticals (Turck *et al.*, 1996), dyes (Rajagopal & Seshadri, 1990) and fungicides. In continuation of our investigation of the chemistry of 1,2-benzothiazine 1,1-dioxide derivatives (Rehman *et al.*, 2005; Rehman *et al.*, 2006) we have synthesized the title compound (I) and its crystal structure is reported here.

In (I) (Fig. 1), the benzene ring of the benzothiazine nucleus is planar (the maximum least square deviation from the plane of the atoms involved is 0.01 Å) while the thiazine ring adopts a distorted half chair conformation. N1 has a pyramidal geometry projecting the methyl group approximately perpendicular to the thiazine ring. Atoms O1, O3 and O5 lie approximately in the plane of the ring while O2 lies almost perpendicular to it.

The C7—O3 bond length in (I) is longer [1.389 (3)] than in the related molecules having no substitution at O3 [1.352 (9) Å; Golič & Leban, 1987; 1.350 (9) Å; Reck *et al.*, 1988].

C9—O4 bond length [1.201 (13) Å] is observed to be shorter than in its previously reported non acylated analogue [1.262 (10) Å; Golič & Leban, 1987] due to no involvement of O4 electrons in the hydrogen bonding. O4 lies almost perpendicular to the thiazine ring and the bond angle C7—C8—C9 [127.3 (2) Å] is greater than observed in the related hydrogen bonded oxicams [121.0 (3) Å; Kojić-Prodić & Rużić-Toroš, 1982; 120.9 (2) Å, Fabiola *et al.*, 1998]. Molecules are linked by C—H···O hydrogen bonds (Table1) forming a chain along *a* axis.

### Experimental

Acetyl chloride (1.57 g; 10 mmol) was slowly added to a mixture of methyl 4-hydroxy-2-methyl-2*H*-1,2-benzothiazine-3-carboxylate1,1-dioxide (1.345 g; 5 mmol), triethylamine (0.71 g; 7 mmol) and carbon tetrachloride (25 ml) under nitrogen atmosphere at 273 K. The mixture was stirred for a period of three hours at room temperature and the solvent was evaporated under vacuum. A residue was poured over ice-water mixture to get the white coloured product which was washed with cold water and recrystallized from chloroform-methanol mixture (1:1). Yield 1.31 g; 84°; m.p. 422 K.

### Crystal data

**Table:**

C_13_H_13_NO_6_S	*F*(000) = 648
*M**_r_* = 311.30	*D*_x_ = 1.512 Mg m^−^^3^
Monoclinic, *P*2_1_/*c*	Mo *K*α radiation, λ = 0.71073 Å
Hall symbol: -P 2ybc	Cell parameters from 15145 reflections
*a* = 6.8917 (5) Å	θ = 2.9–27.5°
*b* = 24.1814 (17) Å	µ = 0.26 mm^−^^1^
*c* = 8.2861 (5) Å	*T* = 120 K
β = 97.876 (4)°	Block, colourless
*V* = 1367.86 (16) Å^3^	0.40 × 0.20 × 0.20 mm
*Z* = 4	

### Data collection

**Table:**

Bruker Nonius CCD camera on κ-goniostat diffractometer	3032 independent reflections
Radiation source: Bruker Nonius FR591 Rotating Anode	2183 reflections with *I* > 2σ(*I*)
graphite	*R*_int_ = 0.057
Detector resolution: 9.091 pixels mm^-1^	θ_max_ = 27.5°, θ_min_ = 3.0°
φ and ω scans to fill the asymmetric unit	*h* = −8→8
Absorption correction: multi-scan (*SADABS*; Sheldrick, 2007)	*k* = −31→30
*T*_min_ = 0.902, *T*_max_ = 0.949	*l* = −10→9
12265 measured reflections	

### Refinement

**Table:**

Refinement on *F*^2^	Primary atom site location: structure-invariant direct methods
Least-squares matrix: full	Secondary atom site location: difference Fourier map
*R*[*F*^2^ > 2σ(*F*^2^)] = 0.050	Hydrogen site location: inferred from neighbouring sites
*wR*(*F*^2^) = 0.145	H-atom parameters constrained
*S* = 1.03	*w* = 1/[σ^2^(*F*_o_^2^) + (0.0764*P*)^2^ + 0.6225*P*] where *P* = (*F*_o_^2^ + 2*F*_c_^2^)/3
3032 reflections	(Δ/σ)_max_ = 0.001
193 parameters	Δρ_max_ = 0.36 e Å^−^^3^
0 restraints	Δρ_min_ = −0.55 e Å^−^^3^

### Special details

**Table:**

Experimental. *SADABS* was used to perform the Absorption correction Estimated minimum and maximum transmission: 0.6195 0.7456 The given Tmin and Tmax were generated using the *SHELX* SIZE command
Geometry. All e.s.d.'s (except the e.s.d. in the dihedral angle between two l.s. planes) are estimated using the full covariance matrix. The cell e.s.d.'s are taken into account individually in the estimation of e.s.d.'s in distances, angles and torsion angles; correlations between e.s.d.'s in cell parameters are only used when they are defined by crystal symmetry. An approximate (isotropic) treatment of cell e.s.d.'s is used for estimating e.s.d.'s involving l.s. planes.
Refinement. Refinement of *F*^2^ against ALL reflections. The weighted *R*-factor *wR* and goodness of fit *S* are based on *F*^2^, conventional *R*-factors *R* are based on *F*, with *F* set to zero for negative *F*^2^. The threshold expression of *F*^2^ > σ(*F*^2^) is used only for calculating *R*-factors(gt) *etc*. and is not relevant to the choice of reflections for refinement. *R*-factors based on *F*^2^ are statistically about twice as large as those based on *F*, and *R*- factors based on ALL data will be even larger.

### Fractional atomic coordinates and isotropic or equivalent isotropic displacement parameters (Å^2^)

**Table:**

	*x*	*y*	*z*	*U*_iso_*/*U*_eq_	
C1	0.5172 (4)	0.43090 (10)	0.7387 (3)	0.0183 (5)	
C2	0.7043 (4)	0.45347 (10)	0.7686 (3)	0.0213 (6)	
H2	0.7629	0.4616	0.8766	0.026*	
C3	0.8032 (4)	0.46376 (10)	0.6369 (3)	0.0213 (5)	
H3	0.9315	0.4790	0.6544	0.026*	
C4	0.7153 (4)	0.45192 (10)	0.4788 (3)	0.0219 (6)	
H4	0.7836	0.4597	0.3893	0.026*	
C5	0.5299 (4)	0.42895 (9)	0.4508 (3)	0.0183 (5)	
H5	0.4719	0.4211	0.3425	0.022*	
C6	0.4276 (4)	0.41729 (9)	0.5809 (3)	0.0167 (5)	
C7	0.2364 (3)	0.39001 (9)	0.5570 (3)	0.0162 (5)	
C8	0.1613 (4)	0.36162 (10)	0.6749 (3)	0.0181 (5)	
C9	−0.0253 (4)	0.32911 (10)	0.6577 (3)	0.0202 (6)	
C10	−0.2808 (4)	0.28845 (11)	0.4788 (3)	0.0301 (6)	
H10A	−0.2544	0.2510	0.5215	0.045*	
H10B	−0.3298	0.2865	0.3622	0.045*	
H10C	−0.3793	0.3061	0.5364	0.045*	
C11	0.3594 (4)	0.30948 (11)	0.8954 (3)	0.0294 (7)	
H11A	0.2711	0.2784	0.8643	0.044*	
H11B	0.3914	0.3107	1.0143	0.044*	
H11C	0.4800	0.3048	0.8464	0.044*	
C12	0.1291 (4)	0.35858 (10)	0.2862 (3)	0.0207 (6)	
C13	−0.0222 (4)	0.37189 (12)	0.1456 (3)	0.0307 (7)	
H13A	−0.0128	0.3455	0.0572	0.046*	
H13B	−0.0008	0.4094	0.1073	0.046*	
H13C	−0.1526	0.3695	0.1796	0.046*	
N1	0.2622 (3)	0.36187 (8)	0.8366 (2)	0.0185 (5)	
O1	0.4912 (3)	0.41115 (8)	1.04687 (19)	0.0263 (4)	
O2	0.2249 (3)	0.46261 (7)	0.8823 (2)	0.0234 (4)	
O3	0.1272 (2)	0.39845 (6)	0.40526 (18)	0.0184 (4)	
O4	−0.0967 (3)	0.31203 (8)	0.7727 (2)	0.0353 (5)	
O5	−0.1014 (3)	0.32064 (7)	0.5028 (2)	0.0266 (4)	
O6	0.2420 (3)	0.32061 (7)	0.3014 (2)	0.0282 (5)	
S1	0.37225 (9)	0.42031 (2)	0.89441 (7)	0.0197 (2)	

### Atomic displacement parameters (Å^2^)

**Table:**

	*U*^11^	*U*^22^	*U*^33^	*U*^12^	*U*^13^	*U*^23^
C1	0.0191 (13)	0.0190 (12)	0.0169 (12)	0.0010 (10)	0.0030 (10)	0.0017 (9)
C2	0.0201 (14)	0.0197 (12)	0.0222 (13)	−0.0006 (10)	−0.0035 (10)	−0.0003 (10)
C3	0.0138 (13)	0.0196 (12)	0.0302 (14)	−0.0010 (10)	0.0021 (10)	0.0026 (11)
C4	0.0205 (14)	0.0207 (12)	0.0260 (13)	0.0023 (10)	0.0082 (10)	0.0032 (10)
C5	0.0193 (14)	0.0183 (12)	0.0175 (12)	0.0029 (10)	0.0034 (10)	0.0009 (9)
C6	0.0162 (13)	0.0149 (11)	0.0182 (12)	0.0021 (9)	−0.0003 (10)	0.0017 (9)
C7	0.0146 (12)	0.0198 (12)	0.0135 (11)	0.0019 (10)	−0.0006 (9)	−0.0019 (9)
C8	0.0204 (14)	0.0183 (12)	0.0151 (11)	0.0004 (10)	0.0009 (9)	−0.0017 (9)
C9	0.0214 (14)	0.0197 (12)	0.0201 (13)	0.0000 (10)	0.0047 (10)	−0.0040 (10)
C10	0.0250 (16)	0.0325 (15)	0.0320 (15)	−0.0132 (12)	0.0013 (12)	−0.0068 (12)
C11	0.0372 (17)	0.0243 (14)	0.0257 (14)	0.0046 (12)	0.0006 (12)	0.0038 (11)
C12	0.0186 (14)	0.0267 (13)	0.0175 (12)	−0.0040 (11)	0.0046 (10)	−0.0015 (10)
C13	0.0280 (16)	0.0437 (17)	0.0190 (13)	−0.0027 (13)	−0.0015 (11)	−0.0039 (12)
N1	0.0192 (12)	0.0202 (10)	0.0157 (10)	−0.0012 (8)	0.0013 (8)	0.0007 (8)
O1	0.0243 (11)	0.0390 (11)	0.0141 (9)	−0.0034 (8)	−0.0023 (7)	0.0010 (8)
O2	0.0248 (10)	0.0230 (9)	0.0223 (9)	0.0011 (7)	0.0031 (7)	−0.0041 (7)
O3	0.0176 (9)	0.0218 (9)	0.0152 (8)	0.0003 (7)	−0.0003 (7)	−0.0008 (7)
O4	0.0386 (13)	0.0458 (12)	0.0225 (10)	−0.0196 (10)	0.0080 (9)	0.0008 (9)
O5	0.0222 (10)	0.0351 (10)	0.0223 (9)	−0.0119 (8)	0.0020 (7)	−0.0044 (8)
O6	0.0308 (11)	0.0284 (10)	0.0259 (10)	0.0028 (8)	0.0054 (8)	−0.0049 (8)
S1	0.0207 (4)	0.0234 (3)	0.0147 (3)	−0.0017 (2)	0.0014 (2)	−0.0012 (2)

### Geometric parameters (Å, °)

**Table:**

C1—C2	1.391 (3)	C10—O5	1.451 (3)
C1—C6	1.407 (3)	C10—H10A	0.9800
C1—S1	1.755 (3)	C10—H10B	0.9800
C2—C3	1.386 (4)	C10—H10C	0.9800
C2—H2	0.9500	C11—N1	1.484 (3)
C3—C4	1.396 (3)	C11—H11A	0.9800
C3—H3	0.9500	C11—H11B	0.9800
C4—C5	1.384 (3)	C11—H11C	0.9800
C4—H4	0.9500	C12—O6	1.199 (3)
C5—C6	1.396 (3)	C12—O3	1.380 (3)
C5—H5	0.9500	C12—C13	1.488 (3)
C6—C7	1.463 (3)	C13—H13A	0.9800
C7—C8	1.353 (3)	C13—H13B	0.9800
C7—O3	1.389 (3)	C13—H13C	0.9800
C8—N1	1.422 (3)	N1—S1	1.644 (2)
C8—C9	1.498 (3)	O1—S1	1.4258 (17)
C9—O4	1.203 (3)	O2—S1	1.4354 (18)
C9—O5	1.334 (3)		
			
C2—C1—C6	122.4 (2)	O5—C10—H10C	109.5
C2—C1—S1	122.25 (18)	H10A—C10—H10C	109.5
C6—C1—S1	115.33 (18)	H10B—C10—H10C	109.5
C3—C2—C1	118.3 (2)	N1—C11—H11A	109.5
C3—C2—H2	120.8	N1—C11—H11B	109.5
C1—C2—H2	120.8	H11A—C11—H11B	109.5
C2—C3—C4	120.4 (2)	N1—C11—H11C	109.5
C2—C3—H3	119.8	H11A—C11—H11C	109.5
C4—C3—H3	119.8	H11B—C11—H11C	109.5
C5—C4—C3	120.8 (2)	O6—C12—O3	121.9 (2)
C5—C4—H4	119.6	O6—C12—C13	128.5 (2)
C3—C4—H4	119.6	O3—C12—C13	109.5 (2)
C4—C5—C6	120.3 (2)	C12—C13—H13A	109.5
C4—C5—H5	119.9	C12—C13—H13B	109.5
C6—C5—H5	119.9	H13A—C13—H13B	109.5
C5—C6—C1	117.9 (2)	C12—C13—H13C	109.5
C5—C6—C7	121.9 (2)	H13A—C13—H13C	109.5
C1—C6—C7	120.2 (2)	H13B—C13—H13C	109.5
C8—C7—O3	121.0 (2)	C8—N1—C11	116.50 (18)
C8—C7—C6	123.9 (2)	C8—N1—S1	115.14 (15)
O3—C7—C6	114.97 (19)	C11—N1—S1	117.95 (17)
C7—C8—N1	119.5 (2)	C12—O3—C7	119.17 (18)
C7—C8—C9	127.3 (2)	C9—O5—C10	115.4 (2)
N1—C8—C9	113.2 (2)	O1—S1—O2	119.20 (10)
O4—C9—O5	124.0 (2)	O1—S1—N1	108.13 (11)
O4—C9—C8	123.0 (2)	O2—S1—N1	107.37 (10)
O5—C9—C8	113.0 (2)	O1—S1—C1	110.97 (12)
O5—C10—H10A	109.5	O2—S1—C1	108.25 (11)
O5—C10—H10B	109.5	N1—S1—C1	101.39 (11)
H10A—C10—H10B	109.5		
			
C6—C1—C2—C3	1.3 (4)	C7—C8—N1—C11	−108.8 (3)
S1—C1—C2—C3	−175.79 (18)	C9—C8—N1—C11	72.3 (3)
C1—C2—C3—C4	0.4 (4)	C7—C8—N1—S1	35.6 (3)
C2—C3—C4—C5	−1.1 (4)	C9—C8—N1—S1	−143.34 (17)
C3—C4—C5—C6	0.1 (4)	O6—C12—O3—C7	−10.5 (3)
C4—C5—C6—C1	1.6 (3)	C13—C12—O3—C7	170.6 (2)
C4—C5—C6—C7	−176.3 (2)	C8—C7—O3—C12	−86.4 (3)
C2—C1—C6—C5	−2.3 (3)	C6—C7—O3—C12	98.0 (2)
S1—C1—C6—C5	175.01 (17)	O4—C9—O5—C10	−0.2 (4)
C2—C1—C6—C7	175.6 (2)	C8—C9—O5—C10	178.9 (2)
S1—C1—C6—C7	−7.1 (3)	C8—N1—S1—O1	−170.76 (17)
C5—C6—C7—C8	157.1 (2)	C11—N1—S1—O1	−27.0 (2)
C1—C6—C7—C8	−20.7 (4)	C8—N1—S1—O2	59.42 (19)
C5—C6—C7—O3	−27.4 (3)	C11—N1—S1—O2	−156.78 (18)
C1—C6—C7—O3	154.8 (2)	C8—N1—S1—C1	−54.01 (19)
O3—C7—C8—N1	−169.13 (19)	C11—N1—S1—C1	89.79 (19)
C6—C7—C8—N1	6.2 (4)	C2—C1—S1—O1	−28.5 (2)
O3—C7—C8—C9	9.6 (4)	C6—C1—S1—O1	154.23 (17)
C6—C7—C8—C9	−175.1 (2)	C2—C1—S1—O2	104.1 (2)
C7—C8—C9—O4	−168.4 (3)	C6—C1—S1—O2	−73.2 (2)
N1—C8—C9—O4	10.4 (3)	C2—C1—S1—N1	−143.1 (2)
C7—C8—C9—O5	12.4 (4)	C6—C1—S1—N1	39.6 (2)
N1—C8—C9—O5	−168.8 (2)		

### Hydrogen-bond geometry (Å, °)

**Table:**

*D*—H···*A*	*D*—H	H···*A*	*D*···*A*	*D*—H···*A*
C13—H13A···O4^i^	0.98	2.49	3.387 (3)	153
C5—H5···O1^i^	0.95	2.48	3.349 (3)	151

Symmetry codes: (i) *x*, *y*, *z*−1.
